# Alleviation of neuropathic pain with neuropeptide Y requires spinal *Npy1r* interneurons that coexpress *Grp*

**DOI:** 10.1172/jci.insight.169554

**Published:** 2023-11-22

**Authors:** Tyler S. Nelson, Heather N. Allen, Paramita Basu, Pranav Prasoon, Eileen Nguyen, Cynthia M. Arokiaraj, Diogo F.S. Santos, Rebecca P. Seal, Sarah E. Ross, Andrew J. Todd, Bradley K. Taylor

**Affiliations:** 1 Department of Anesthesiology and Perioperative Medicine,; 2 Pittsburgh Project to end Opioid Misuse,; 3 Center for Neuroscience,; 4 Pittsburgh Center for Pain Research, and; 5 Department of Neurobiology, University of Pittsburgh School of Medicine, Pittsburgh, Pennsylvania, USA.; 6 Spinal Cord Group, School of Psychology and Neuroscience, University of Glasgow, Glasgow, United Kingdom.

**Keywords:** Neuroscience, NPY, Pain, Pharmacology

## Abstract

Neuropeptide Y targets the Y1 receptor (Y1) in the spinal dorsal horn (DH) to produce endogenous and exogenous analgesia. DH interneurons that express Y1 (Y1-INs; encoded by *Npy1r*) are necessary and sufficient for neuropathic hypersensitivity after peripheral nerve injury. However, as Y1-INs are heterogenous in composition in terms of morphology, neurophysiological characteristics, and gene expression, we hypothesized that a more precisely defined subpopulation mediates neuropathic hypersensitivity. Using fluorescence in situ hybridization, we found that Y1-INs segregate into 3 largely nonoverlapping subpopulations defined by the coexpression of *Npy1r* with gastrin-releasing peptide (*Grp*/*Npy1r*), neuropeptide FF (*Npff*/*Npy1r*), and cholecystokinin (*Cck*/*Npy1r*) in the superficial DH of mice, nonhuman primates, and humans. Next, we analyzed the functional significance of *Grp*/*Npy1r*, *Npff*/*Npy1r*, and *Cck*/*Npy1r* INs to neuropathic pain using a mouse model of peripheral nerve injury. We found that chemogenetic inhibition of *Npff*/*Npy1r*-INs did not change the behavioral signs of neuropathic pain. Further, inhibition of Y1-INs with an intrathecal Y1 agonist, [Leu^31^, Pro^34^]-NPY, reduced neuropathic hypersensitivity in mice with conditional deletion of *Npy1r* from CCK-INs and NPFF-INs but not from GRP-INs. We conclude that *Grp*/*Npy1r*-INs are conserved in higher order mammalian species and represent a promising and precise pharmacotherapeutic target for the treatment of neuropathic pain.

## Graphical abstract

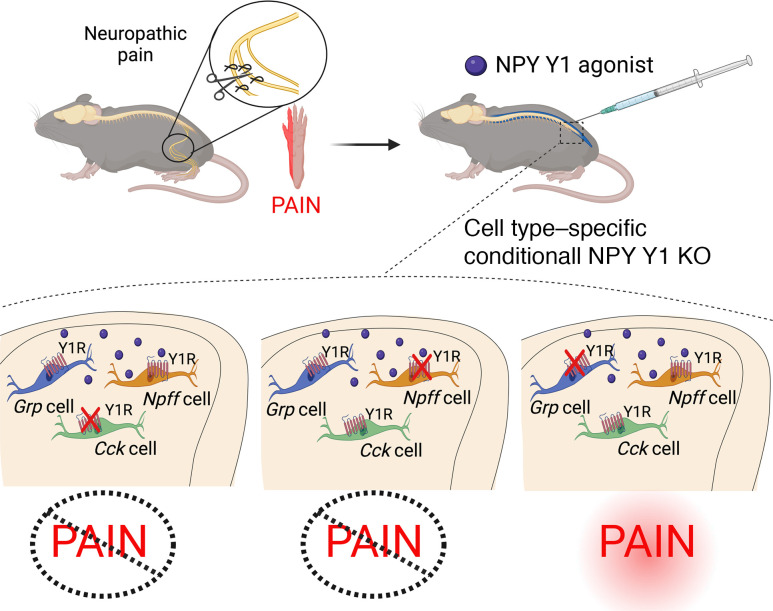

## Introduction

Pain protects organisms from injury, and neurons in the superficial spinal cord dorsal horn (DH) are fundamental for the processing of harmful or potentially harmful nociceptive input (1). Most DH interneurons (INs) are glutamatergic with marked functional and chemical heterogeneity (2–4). These glutamatergic DH neurons mediate the appropriate and maladaptive expression of pain behavior (2, 5–7). For example, peripheral nerve damage can lead to pathological allodynia in which normally innocuous sensory input is amplified by excitatory DH neurons and conveyed as painful (7–10). The molecular identification of the subpopulations of excitatory DH neurons that mediate allodynia is an intense area of investigation (9), including those that express protein kinase C γ (PKCγ) (11–18), somatostatin (SST) (19, 20), cholecystokinin (CCK) (14, 21), neurokinin 1 receptor (NK1R) (22), mu opiate receptor (OPRM1) (23), and neuropeptide Y Y1 receptor (NPY1R) (24–26). Among these, *Npy1r*-expressing interneurons (Y1-INs) represent a particularly promising, druggable, pharmacotherapeutic target for the treatment of neuropathic pain (24, 26). We recently demonstrated that Y1-INs are both necessary and sufficient for the manifestation of neuropathic pain-like behavior using cell type–specific modulation/ablation studies (24, 25). Importantly, the Y1 receptor is coupled to inhibitory G proteins (G_i/o_), and application of NPY Y1–selective agonists reduces the excitability of Y1-INs and decreases pro-nociceptive signaling both in vitro and in vivo (27–30). Markedly, intrathecal administration of Y1 agonist efficaciously reduces behavioral signs of neuropathic pain, and this can be reversed with Y1-selective antagonists (24, 31–33). These findings establish Y1 on Y1-INs as the general target for the antihyperalgesic effects of NPY.

However, further questions are raised by the highly heterogenous composition of Y1-INs in terms of morphology, neurophysiological characteristics, and gene expression (25, 26, 34, 35). For example, Y1 immunoreactivity in the rat revealed distinctive morphological and neurochemical Y1-IN subpopulations (25, 34, 35), our ex vivo lumbar spinal cord slice recordings from a reliable *Npy1r*^eGFP^ mouse line identified 4 distinct neurophysiological firing patterns of Y1-INs (24, 29), and unbiased single-cell transcriptomics in the mouse identified *Npy1r* expression in 3 excitatory DH neuron clusters (36). The heterogeneity of Y1-INs may suggest the existence of subpopulations with distinct physiological roles. Of particular interest are recent results that segregate excitatory interneurons into largely nonoverlapping populations defined by the expression of CCK, neurotensin, neurokinin B (NKB), neuropeptide FF (NPFF), substance P (SP; encoded by *Tac1*), enhanced green fluorescent protein (EGFP) or Cre recombinase under control of the gastrin-releasing peptide (*Grp*) promoter in BAC transgenic mice from the GENSAT project (*Grp*^eGFP^ and *Grp*^Cre^), and gastrin-releasing peptide receptor (GRPR) (37–40). Here, we performed fluorescence in situ hybridization (FISH) to characterize the coexpression of these markers with the *Npy1r* gene. We found that Y1-INs largely segregated into 3 subpopulations (demarcated by the coexpression of *Npy1r* with *Cck*, *Grp*, and *Npff*, respectively), which are conserved in the nonhuman primate and human DH. We then determined the functional contribution of the *Npy1r/Npff* population to the behavioral manifestation of mechanical and cold allodynia after peripheral nerve injury. Further, we asked whether pharmacological inhibition with a NPY Y1 agonist eliminates the signs of neuropathic pain in mice with conditional deletion of *Npy1r* in GRP-INs, CCK-INs, or NPFF-INs. Together, our results elucidate the molecular makeup and functional role of Y1-IN subpopulations and support *Grp/Npy1r*-INs as an optimal, evolutionarily conserved, and relatively precise pharmacotherapeutic target for the treatment of neuropathic pain.

## Results

### Y1-INs are excitatory neurons that segregate into 3 distinct subpopulations.

**Figure 1:**
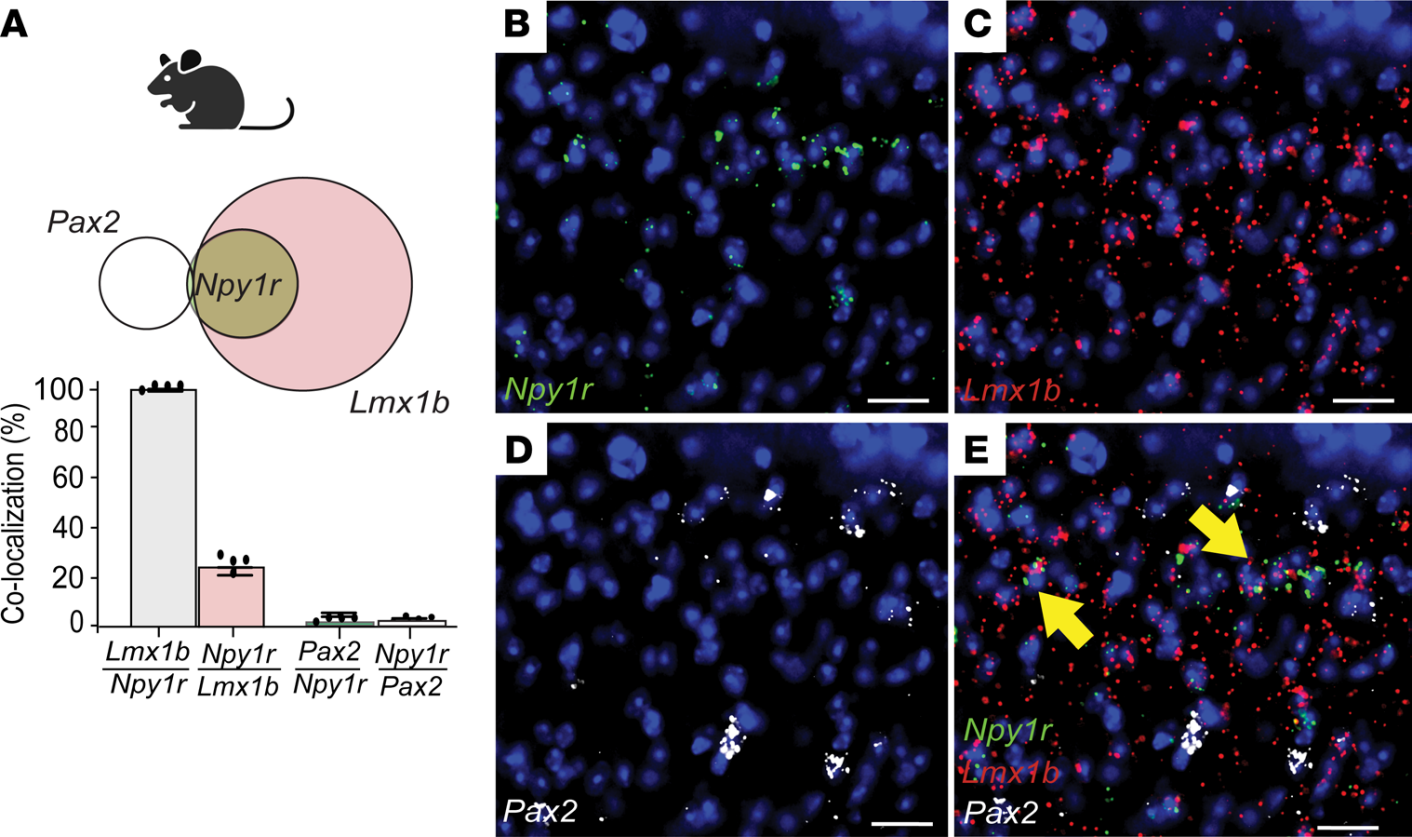
Y1-INs are a glutamatergic subpopulation in mouse lumbar superficial dorsal horn. (**A**–**E**) *Npy1r* in laminae I-II extensively colocalizes with *Lmx1b* (*Lmx1b*/*Npy1r* 96.91% ± 0.49%; *Npy1r*/*Lmx1b* 25.18% ± 1.59%) but not *Pax2* (*Pax2*/*Npy1r* 1.39% ± 0.47%; *Npy1r*/*Pax2* 2.17% ± 0.81%) (*n* = 4–5 mice/group). Each data point indicates the average of 2–4 quantified sections/mouse. Scale bars: 25 μm. Yellow arrows indicate colocalization. Data shown as mean ± SEM.

**Figure 2:**
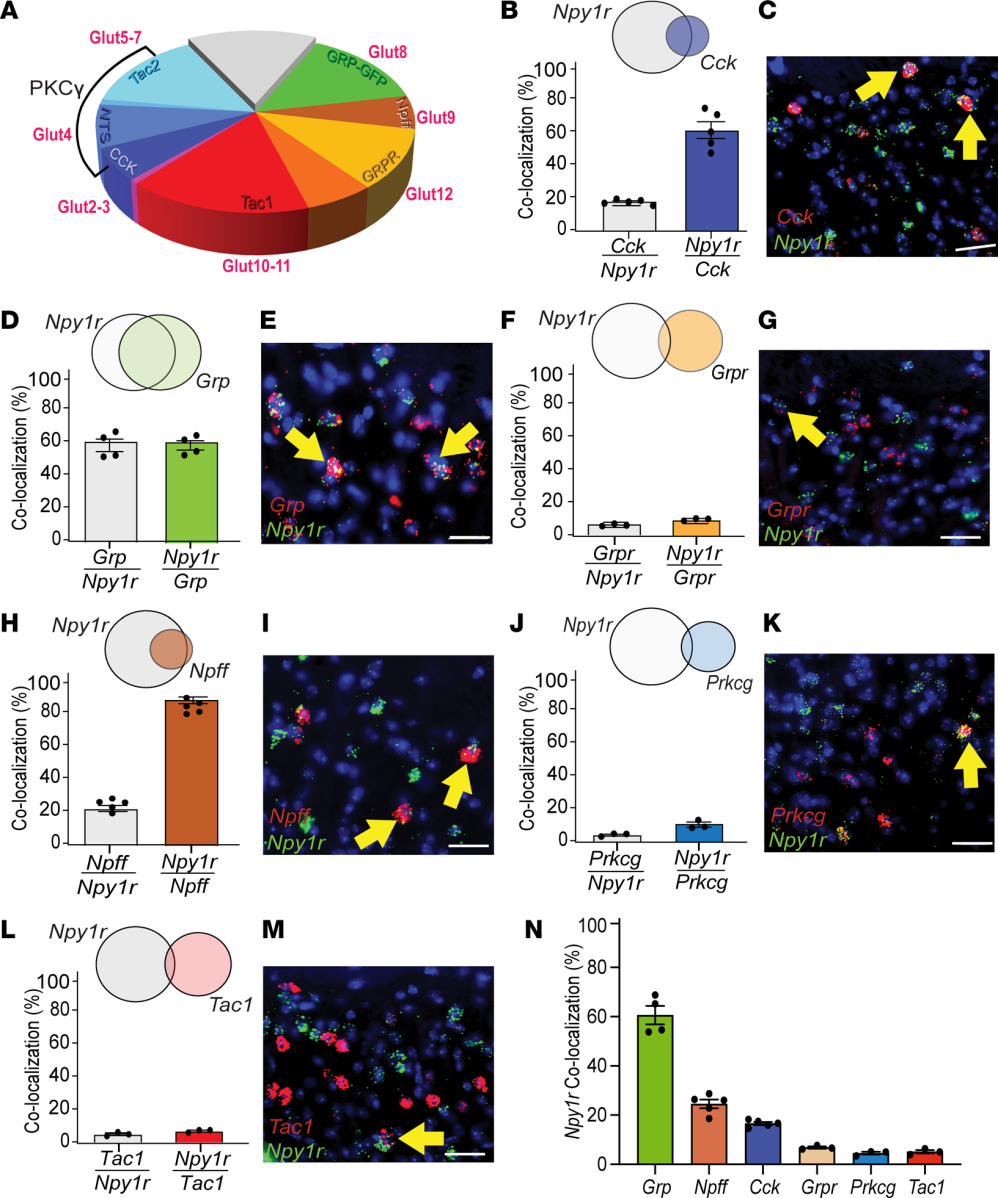
Identification of major subpopulations of Y1-INs in mouse lumbar superficial dorsal horn. (**A**) Excitatory interneurons segregate into largely nonoverlapping subpopulations defined by expression of cholecystokinin (CCK), neurotensin, neurokinin B (NKB), neuropeptide FF (NPFF), substance P (SP; encoded by *Tac1*), enhanced green fluorescent protein (EGFP) or Cre recombinase under control of the gastrin-releasing peptide (*Grp*) promoter in BAC transgenic mice from the GENSAT project (*Grp*^eGFP^ and *Grp*^Cre^), and gastrin-releasing peptide receptor (GRPR). (**B**–**N**) *Npy1r* colocalizes with superficial *Cck* (*Cck*/*Npy1r* 16.60% ± 0.63%; *Npy1r*/*Cck* 56.98% ± 4.73%), *Grp* (*Grp*/*Npy1r* 60.61% ± 3.78%; *Npy1r*/*Grp* 60.58% ± 2.78%), and *Npff* (*Npff*/*Npy1r* 24.55% ± 1.75%; *Npy1r*/*Npff* 91.21% ± 2.00%) but not *Grpr* (*Grpr*/*Npy1r* 6.85% ± 0.45%; *Npy1r*/*Grpr* 10.16% ± 0.43%), *Prkcg* (*Prkcg*/*Npy1r* 4.55% ± 0.55%; *Npy1r*/*Prkcg* 10.59% ± 1.50%), or *Tac1* (*Tac1*/*Npy1r* 5.13% ± 0.68%; *Npy1r*/*Tac1* 7.09% ± 0.55%) (*n* = 3–5 mice/group). Each data point indicates the average of 2–4 quantified sections/mouse. Scale bars: 25 μm. Yellow arrows indicate colocalization. Data shown as mean ± SEM.

We used multilabel FISH to neurochemically characterize L4 Y1-INs in superficial DH. First, in mouse, we found *Npy1r* extensively colocalized with the excitatory marker LIM homeobox transcription factor 1-β (*Lmx1b*) (96.91% ± 0.49%) but not the inhibitory marker paired box 2 (*Pax2*) (1.39% ± 0.47%) (Figure 1, A–E) (41–44), indicating that Y1-INs are glutamatergic. Next, we identified putative Y1-IN subpopulation(s) using recent data that segregate approximately 90% of DH INs into largely nonoverlapping excitatory interneuron subpopulations based on the expression of neuropeptides/receptors (36–40, 45, 46) (Figure 2A). We found that *Npy1r* colocalized with *Cck* (16.60% ± 0.63%), *Grp* (60.61% ± 3.78%), and *Npff* (24.55% ± 1.75%) but minimally with *Grpr* (6.85% ± 0.45%), protein kinase C γ (*Prkcg*) (4.55% ± 0.55%), or *Tac1* (5.13% ± 0.68%) (Figure 2, B–N). Note that *Prkcg* was used as a marker to broadly encompass both the neurotensin and NKB subpopulations (Figure 2A). Next, we characterized DH *Npy1r* expression with canonical genes implicated in neuropathic and inflammatory mechanical allodynia (9, 14, 19, 20, 22, 47) and found that *Npy1r* colocalized extensively with *Sst* (49.26% ± 0.98%) and *Calb2* (33.96% ± 2.42%) but not *Tacr1* (6.94% ± 1.11%) (Supplemental Figure 1, A–F; supplemental material available online with this article; https://doi.org/10.1172/jci.insight.169554DS1). Last, recent single-cell RNA-sequencing data sets have suggested 2 genes, *Nmur2* and *Car12*, have significant overlap with *Npy1r* (36, 45). *Nmur2* encodes the neuromedin U receptor type 2 and contributes to nociceptive processing (48), and *Car12* encodes the enzyme carbonic anhydrase 12 that is highly enriched in dorsal horn somatostatin neurons (49) but has no identified role in pain processing. Here, we verified the colocalization of *Npy1r* with both *Nmur2* (37.04% ± 0.94%) and *Car12* (32.83% ± 6.33%) (Supplemental Figure 1, G–J). Together, these results indicate that Y1-INs are excitatory DH interneurons and largely segregate into 3 largely nonoverlapping subpopulations demarcated by coexpression of *Npy1r* with the genes *Cck*, *Npff*, or *Grp*. Further, many Y1-INs colocalize with *Sst*, *Calb2*, *Car12*, and *Nmur2*.

### Y1-IN subpopulations are conserved across higher order mammalian species.

**Figure 3:**
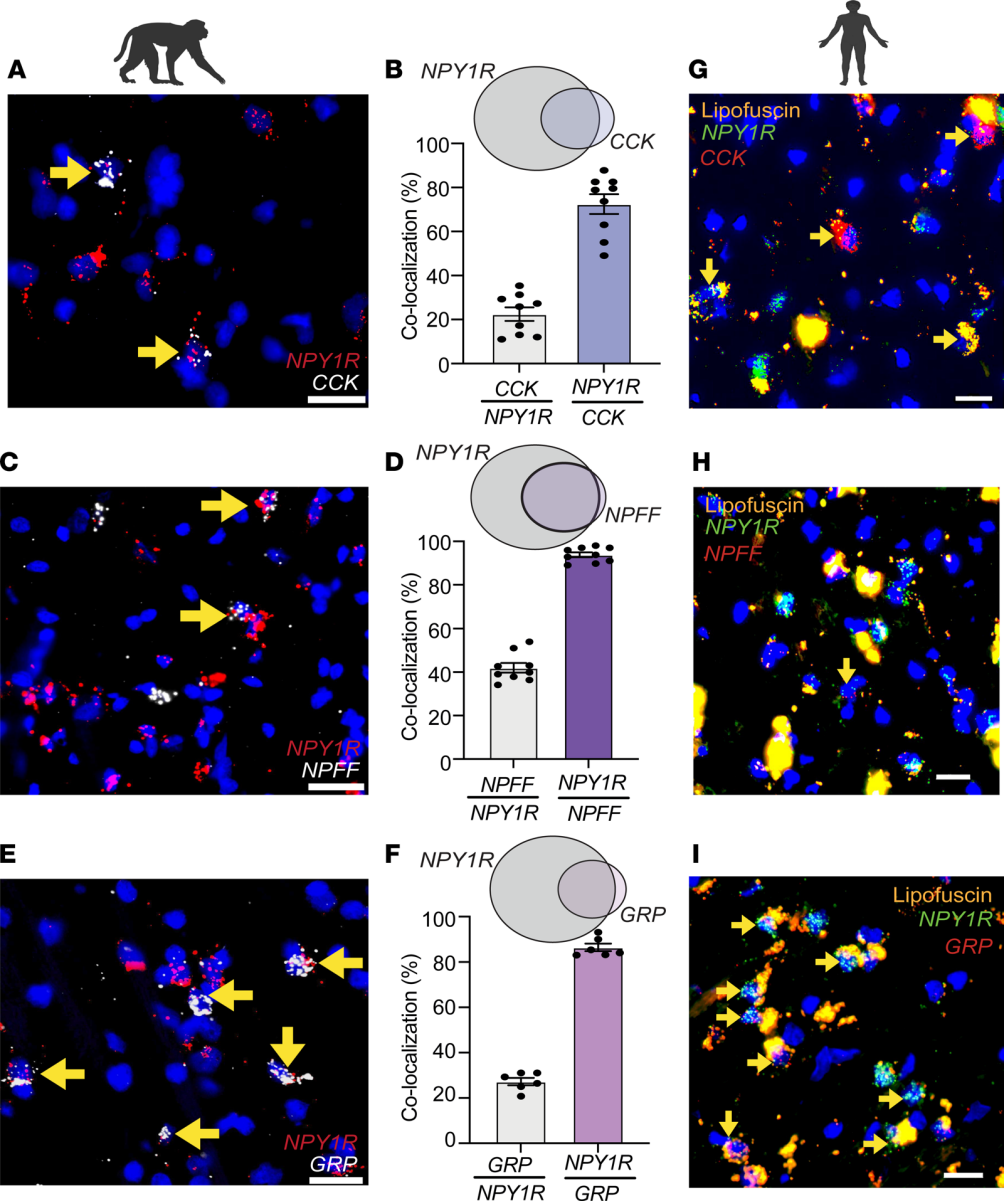
Conservation of *NPY1R*/*CCK,* *NPY1R*/*GRP*, and *NPY1R*/*NPFF* subpopulations in the superficial dorsal horn of nonhuman primates and humans. (**A**–**F**) *NPY1R* in the substantia gelatinosa extensively colocalizes with superficial *CCK* (*CCK*/*NPY1R* 22.49% ± 3.08%; *NPY1R*/*CCK* 72.45% ± 4.53%), *GRP* (*GRP*/*NPY1R* 27.26% ± 1.63%; *NPY1R*/*GRP* 86.46% ± 1.70%), and *NPFF* (*NPFF*/*NPY1R* 41.98% ± 2.20%; *NPY1R*/*NPFF* 93.82% ± 1.12%) in the rhesus macaque (*n* = 2 macaques). Individual data points represent 1 single quantified section. Scale bars: 25 μm. (**G**–**I**) *NPY1R* colocalizes with superficial *CCK*, *NPFF*, and *GRP* in the human spinal cord dorsal horns (*n* = 2 human organ donors). Scale bars: 20 μm. Yellow arrows indicate colocalization. Data shown as mean ± SEM.

To determine the evolutionary conservation of Y1-IN subpopulations, we evaluated the expression of *NPY1R*, *CCK*, *NPFF*, and *GRP* in spinal cord tissue from rhesus macaque and human organ donors. As in the mouse, *NPY1R*-expressing cells were abundantly expressed in the superficial DH of macaque and extensively colocalized with *CCK* (22.49% ± 3.08%), *GRP* (27.26% ± 1.63%), and *NPFF* (41.98% ± 2.20%) (Figure 3, A–F). Similarly, *NPY1R*-expressing cells were plentiful in the superficial DH of human spinal cord and coexpressed *CCK*, *GRP*, and *NPFF* (acknowledging that *NPFF* expression is sparse in human DH as compared with mouse or macaque) (Figure 3, G–I). Intense lipofuscin prevented accurate quantification in the human spinal cord.

### Light brush of the hind paw in mice with neuropathic hypersensitivity evokes Fos in DH Grp/Npy1r-INs.

**Figure 4:**
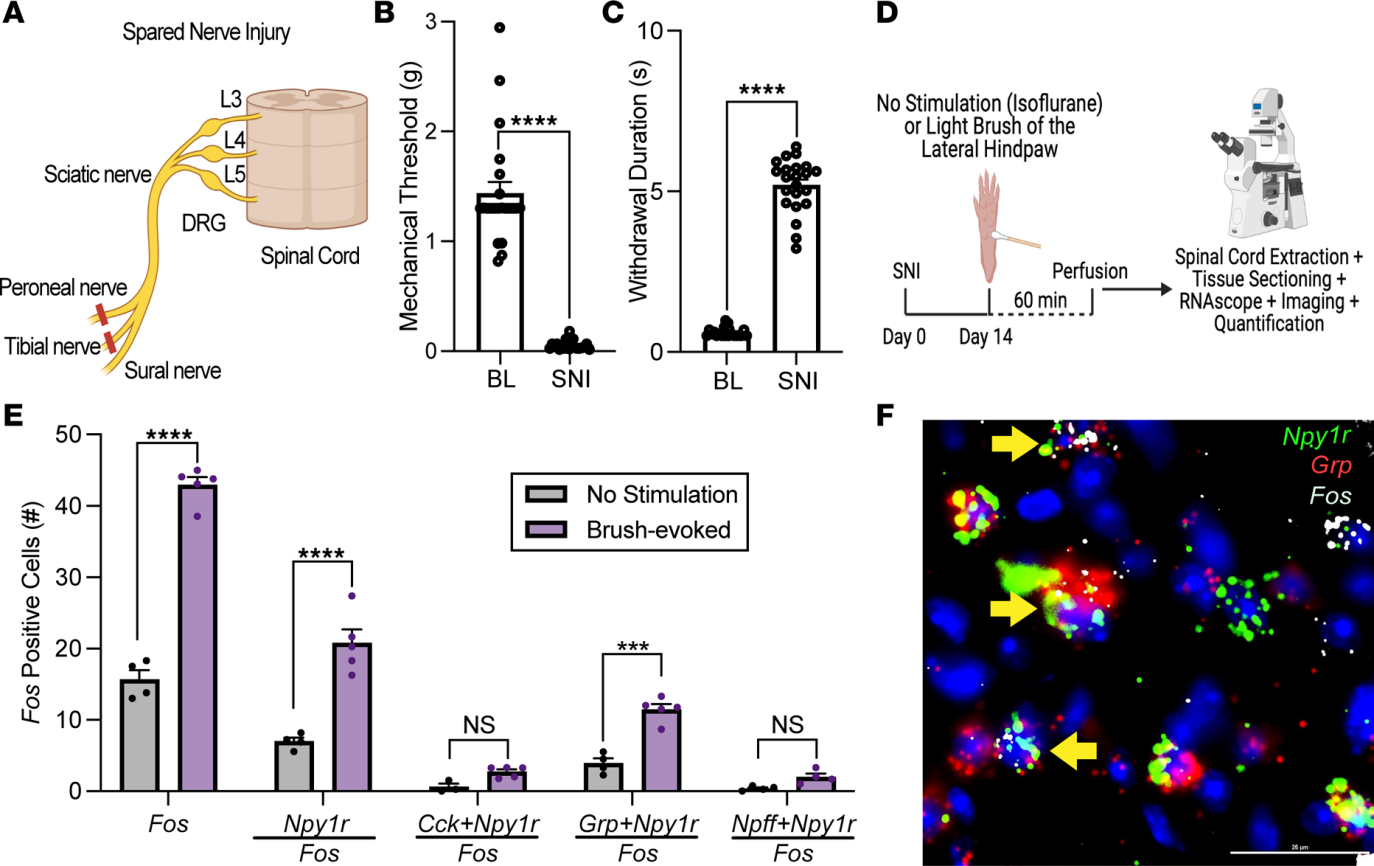
Non-noxious mechanical stimulation increases neuronal activity in *Grp*/*Npy1r*-INs. (**A**) Schematic representation of the spared nerve injury (SNI) model of neuropathic pain. (**B** and **C**) SNI produces mechanical (****P < 0.0001) and cold hypersensitivity (****P < 0.0001) in the ipsilateral hind paw when assessed 14 days after injury. Unpaired 2-tailed *t* test (*n* = 24 mice/group). (**D**) Experimental timeline for SNI, light brush of the lateral hind paw, and FISH labeling for *Fos* in Y1-IN subpopulations in the lumbar dorsal horn of SNI mice. (**E**) Light brush increases *Fos*-positive cells in the superficial dorsal horn (****P < 0.0001) primarily in *Npy1r* neurons (****P < 0.0001) and *Grp*/*Npy1r* neurons (***P = 0.0001) but not *Cck*/*Npy1r* neurons (*P* = 0.7371) or *Npff*/*Npy1r* neurons (*P* = 0.8095) (2-way ANOVA: mRNA expression × stimulation, *F*_4, 33_ = 1.215, *P* < 0.0001, Holm-Šídák post hoc tests) (*n* = 3–5 mice/group). Each data point indicates the average of 2–4 quantified sections/mouse. (**F**) Representative example of *Grp*/*Npy1r*-INs coexpressing light brush–evoked *Fos*. Scale bars: 25 μm. Yellow arrows indicate colocalization. Data shown as mean ± SEM.

Next, we determined whether the primary Y1-IN subpopulation(s) are sensitized by nerve injury. We performed spared nerve injury (SNI), a model of peripheral neuropathic pain (50) (Figure 4A), which produced robust mechanical and cold hypersensitivity in the spared nerve (sural) innervation territory (lateral aspect) of the injured hind paw (Figure 4, B and C) (24, 25). Two weeks after SNI, we applied light brush stimulation to the plantar hind paw (Figure 4D), and as predicted, we found increased *Fos* expression (using the immediate early gene *Fos* as a correlate for neuronal activity) in the ipsilateral superficial DH in *Npy1r*-expressing neurons (Figure 4E). *Fos* expression was principally expressed in the *Grp*/*Npy1r* subpopulation rather than the *Npff*/*Npy1r* or *Cck*/*Npy1r* subpopulations (Figure 4, E and F). These results suggest that in the context of peripheral nerve injury, mechanical stimulation predominately activates Y1-INs that coexpress *Grp*.

### Inhibition of Npff/Npy1r-INs does not change neuropathic hypersensitivity.

**Figure 5:**
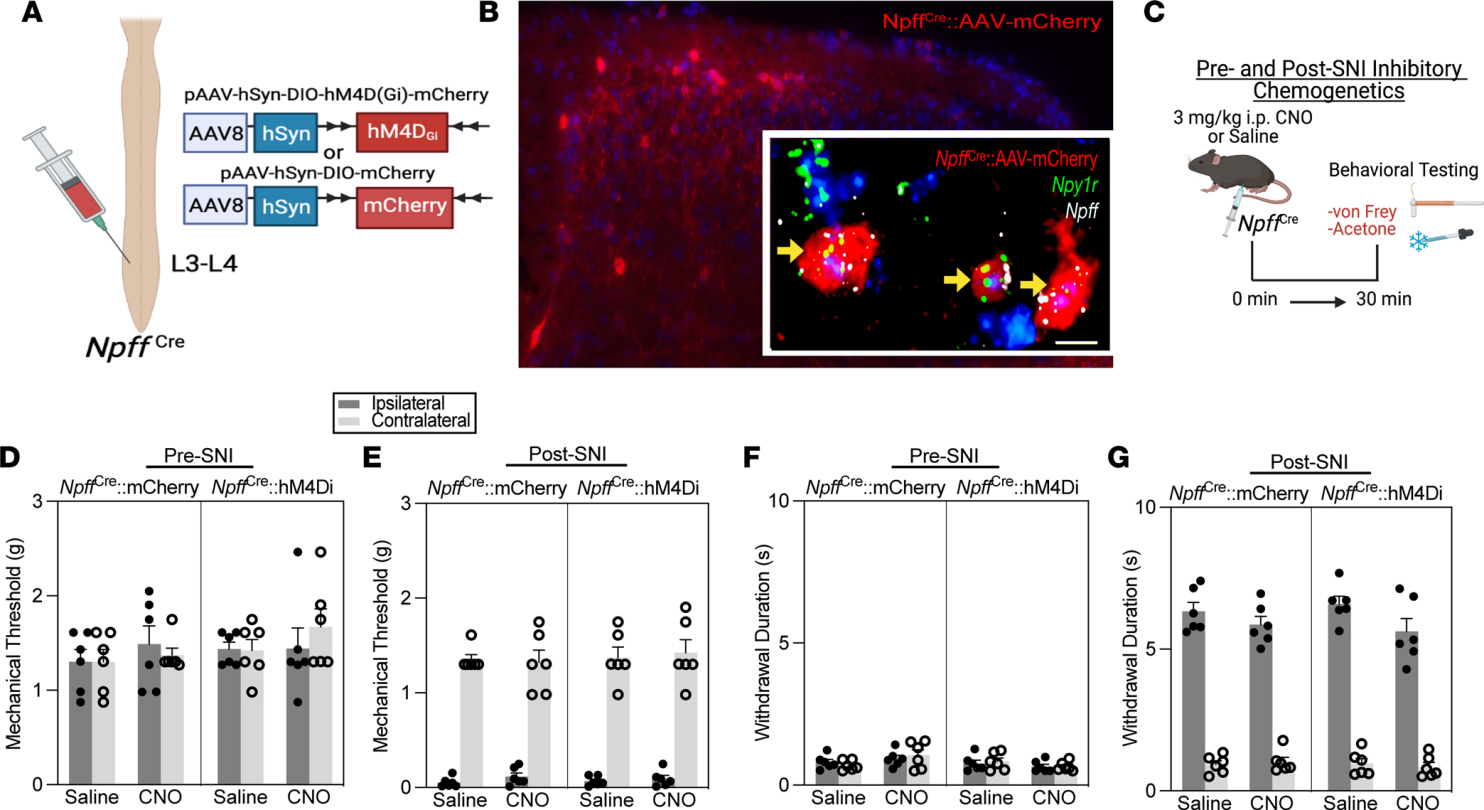
*Npff*/*Npy1r*-INs do not contribute to nerve injury–induced mechanical and cold allodynia. (**A**) Strategy for selectively targeting *Npff*/*Npy1r*-INs in *Npff*^Cre^ mice with intraspinal injections of the Cre-dependent virus AAV8-hSyn-DIO-hM4D_Gi_-mCherry. (**B**) AAV transfection in *Npff*^Cre^ mice (large image) and FISH confirmation of *Npy1r* and *Npff* expression in transfected cells (insert). Scale bar: 10 μm. (**C**) Experimental timeline of chemogenetic reflexive behavioral testing at both pre- and post-SNI time points. (**D** and **E**) Chemogenetic inhibition of NPFF-INs with CNO (3 mg/kg) does not change mechanical sensitivity when conducted either before (3-way repeated measures [RM] ANOVA: drug × virus × side, *F*_1,20_ = 0.7875, *P* = 0.3854) or after SNI (3-way RM ANOVA: drug × virus × side, *F*_1,20_ = 0.6462, *P* = 0.4309) (*n* = 6 mice/group). (**F** and **G**) Chemogenetic inhibition of NPFF-INs with CNO (3 mg/kg) does not change cold sensitivity pre-SNI (3-way RM ANOVA: drug × virus × side, *F*_1,20_ = 1.215, *P* = 0.2834) or post-SNI (3-way RM ANOVA: drug × virus × side, *F*_1,20_ = 0.1615, *P* = 0.6920) (*n* = 6 mice/group). Data shown as mean ± SEM.

Previously, we demonstrated that chemogenetic inhibition of Y1-INs with designer receptors exclusively activated by designer drugs (DREADDs) abolished SNI-induced neuropathic hypersensitivity (24). Similarly, we sought to determine the functional contribution of the *Npff*/*Npy1r*, *Grp*/*Npy1r*, and *Cck*/*Npy1r* subpopulations to neuropathic hypersensitivity. First, we took advantage of the fact that virtually all *Npff*-expressing DH interneurons coexpress *Npy1r* (Figure 2H). This feature of NPFF-INs allowed us to intraspinally administer Cre-dependent inhibitory DREADDs into the left lumbar (targeting L3–L4) DH of our developed *Npff*^Cre^ mouse line to inhibit the activity of *Npff*/*Npy1r*-INs (Figure 5A). As predicted, AAV-mCherry transfection expression with immunohistochemistry and FISH was detected ipsilateral to viral injection (left but not right DH) in superficial DH cells that coexpressed *Npff* and *Npy1r* (Figure 5B). We then intraperitoneally administered a 3 mg/kg dose of clozapine *N*-oxide (CNO, TOCRIS); this dose is the lowest recommended dose to behave as a DREADD agonist in the absence of off-target effects (51) and is commonly utilized for spinal cord chemogenetics (13, 24, 52). Chemogenetic inhibition of *Npff*/*Npy1r*-INs did not change mechanical or cold responses before or after SNI (Figure 5, C–G). These findings indicate that spinal *Npff*/*Npy1r*-INs are not necessary for the behavioral manifestation of mechanical or cold neuropathic hypersensitivity.

### The Y1 agonist [Leu^31^, Pro^34^]-NPY eliminates nerve injury–induced mechanical and cold hypersensitivity in mice with conditional deletion of Cck but not Grp from Npy1r-INs.

**Figure 6:**
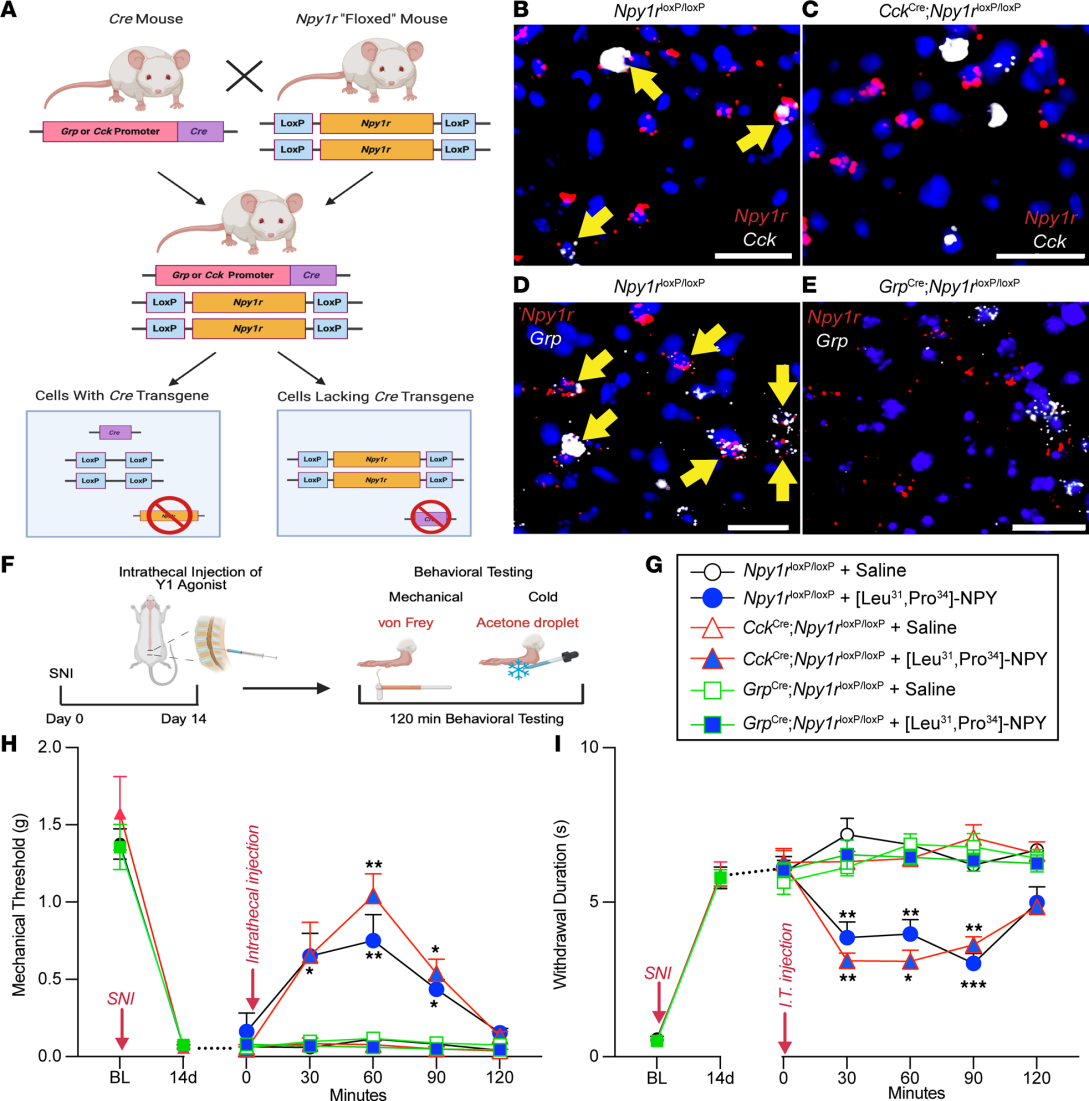
*Grp*/*Npy1r*-INs but not *Cck*/*Npy1r*-INs contribute to the inhibition of nerve injury–induced mechanical and cold allodynia by a Y1 agonist. (**A**) Schematic representation of the conditional genetic knockout breeding protocol to delete *Npy1r* from CCK-Cre and GRP-Cre cells. (**B**–**E**) Confirmation of conditional deletion of *Npy1r*. FISH of sections of the lumbar spinal cord demonstrate that *Npy1r*^loxP/loxP^ mice contain DH neurons that coexpress *Npy1r* and *Cck* as well as *Npy1r* and *Grp*. Conversely, *Npy1r*^loxP/loxP^ *Cck*^Cre^ mice lack expression of *Npy1r* in *Cck*-expressing neurons and *Npy1r*^loxP/loxP^ *Grp*^Cre^ mice lack expression of *Npy1r* in *Grp*-expressing neurons. Yellow arrows indicate colocalization. Scale bars: 25 μm. (**F**) Experimental timeline for SNI, intrathecal pharmacology, and mechanical (von Frey) and cold (acetone droplet withdrawal) behavioral testing. (**G**) Genetic mouse lines and pharmacological drugs represented in **H** and **I**. (**H**) [Leu^31^, Pro^34^]-NPY abolishes SNI-induced mechanical allodynia in *Npy1r*^loxP/loxP^ and *Npy1r*^loxP/loxP^ *Cck*^Cre^ mice but not in *Npy1r*^loxP/loxP^ *Grp*^Cre^ mice (*n* = 8–9 mice/group). Three-way RM ANOVA: time × genotype × drug, *F*_4,128_ = 7.509, *P* < 0.0001; Tukey’s multiple-comparison tests. (**I**) [Leu^31^, Pro^34^]-NPY abolishes SNI-induced cold allodynia in *Npy1r*^loxP/loxP^ and *Npy1r*^loxP/loxP^ *Cck*^Cre^ mice but not in *Npy1r*^loxP/loxP^ *Grp*^Cre^ mice (*n* = 8–9 mice/group). Three-way RM ANOVA: time × genotype × drug, *F*_4,128_ = 6.322, *P* = 0.0001; Tukey’s multiple-comparison tests. *P ≤ 0.05, **P ≤ 0.01, ***P ≤ 0.001. Data shown as mean ± SEM.

In contrast to NPFF-INs, many CCK- and GRP-INs do not coexpress *Npy1r*. As a result, we could not inhibit the *Grp*^Cre^ or *Cck*^Cre^ interneuron populations with a chemogenetic approach as we did with our *Npff*^Cre^ mice because this could also modulate neurons that do not express *Npy1r* (and there is no currently available *Npy1r*^Flp^ mouse line to allow a dual recombinase chemogenetic approach). Instead, we pharmacologically inhibited Y1-INs with an intrathecal Y1 agonist in conditional genetic knockout lines. Specifically, we crossed *Npy1r*^loxP/loxP^ mice (53) with either *Grp*^Cre^ (54) or *Cck*^Cre^ (55) mice to selectively knock out *Npy1r* from *Grp*^Cre^ or *Cck*^Cre^ neurons, respectively (Figure 6, A–E). Next, we performed SNI to produce mechanical and cold hypersensitivity (Figure 6, F–I). Two weeks later, we intrathecally administered the Y1-selective agonist, [Leu^31^, Pro^34^]-NPY, to inhibit spinal Y1-INs (24). [Leu^31^, Pro^34^]-NPY reduced SNI-induced allodynia in both control *Npy1r*^loxP/loxP^ and *Npy1r*^loxP/loxP^ *Cck*^Cre^ mice but not in *Npy1r*^loxP/loxP^ *Grp*^Cre^ mice (Figure 6, F–I). Similarly, intrathecal administration of [Leu^31^, Pro^34^]-NPY reduced SNI-induced allodynia in *Npy1r*^loxP/loxP^ mice crossed with *Npff*^Cre^ mice (Supplemental Figure 2). Conditional genetic knockout of *Npy1r* was confirmed with FISH and quantified for all 3 mouse crosses (Supplemental Figure 3). These results indicate that NPY Y1 agonists inhibit behavioral signs of neuropathic pain by actions at spinal cord *Npy1r*-expressing interneurons that coexpress *Grp* but not *Cck* or *Npff*.

## Discussion

In this study, we demonstrate that spinal *Npy1r*-expressing DH interneurons that coexpress *Grp* are evolutionarily conserved across rodent, nonhuman primate, and human and are necessary for the efficacy of spinally directed Y1 agonists to inhibit neuropathic pain. The *Grp* subpopulation of *Npy1r*-expressing DH INs represent an optimal and precise future pharmacotherapeutic target for the treatment of neuropathic pain.

### Y1-INs segregate into 3 glutamatergic subpopulations.

Consistent with previous results (25, 26, 36, 46), we found that Y1-INs are almost entirely glutamatergic and segregate into 3 largely nonoverlapping excitatory subpopulations. These are demarcated by coexpression of *Npy1r* with *Grp*, *Npff*, or *Cck*, consistent with the 3 excitatory subpopulations (Glut2, Glut8, and Glut9) that Häring et al. identified using single-cell RNA sequencing (36). In that study, *Cck*-expressing neurons fell into 3 separate clusters: Glut1, Glut2, and Glut3. The laminar location of these *Cck*-expressing subpopulations varied with Glut2 neurons scattered in superficial laminae I-II (the same location we found our *Cck*/*Npy1r*-INs), Glut3 neurons forming a compact band in laminae IIi-III, and Glut1 *Cck*-expressing neurons (the largest population) restricted to the deep dorsal horn (laminae III-V) (36, 56). Thus, our *Cck*/*Npy1r*-INs are likely Glut2 neurons with reference to the Häring et al. data set. In the single-cell results, *Npff* was found exclusively in the Glut9 population; thus, our *Npff*/*Npy1r*-INs likely correspond to the Glut9 cluster in the Häring et al. data set (36, 38). In contrast, *Grp* expression was broadly distributed across the Glut5–12 populations in the Häring et al. data set. However, many of these GRP-INs (~30%) are in the Glut8 cluster (exhibiting *Nmur2* and *Reln* expression but not *Npff* expression) (36, 40). Therefore, by process of elimination (lack of effect in *Cck* and *Npff* subpopulations), the *Grp*/*Npy1r*-INs that mediate neuropathic pain and its inhibition by Y1 agonists likely correspond to the Glut8 cluster.

Converging pieces of evidence implicate Glut8 neurons (exhibiting *Nmur2* and *Reln* expression but not *Npff* expression) as both necessary and sufficient for the manifestation of mechanical and thermal hypersensitivity. First, the neuromedin U receptor 2 (encoded by *Nmur2*) is coupled to Gα_q/11_, and intrathecal administration of neuromedin U dose-dependently produces mechanical and heat hypersensitivity (57, 58). Second, Häring et al. found immediate early gene expression in Glut8 neurons in response to both noxious heat and cold stimuli (36). Third, *Reln*-expressing DH neurons also exhibit Fos expression in response to noxious thermal or mechanical stimulation (59). Fourth, recently uncovered ErbB4^+^ neurons that participate in spinal heat signaling are exclusively represented in a *Nmur2*-expressing excitatory neuron subpopulation (60). Last, SST-INs are necessary and sufficient for neuropathic pain (19, 20), and *Sst* expression is also detected in Glut8 neurons (albeit also in other excitatory populations) (36). Thus, we believe that the *Reln*/*Nmur2*/*Sst*/*Grp*/*Npy1r* population in all these studies converges on the Glut8 population and that it is a specific subpopulation that is necessary for neuropathic pain. Therefore, future studies could further investigate the contribution to neuropathic pain of *Npff*-Glut8 neurons (*Reln*^+^, *Nmur2*^+^). Future experiments may perhaps synthesize an *Nmur2*^Cre^ mouse or use an intersectional approach that develops and utilizes a *Npy1r*^Flp^ mouse crossed with a *Grp*^Cre^ mouse to perform more advanced analyses on the Glut8 neuron subpopulation to better define its role in neuropathic pain.

### Grp/Npy1r-expressing DH interneurons mediate the antiallodynic actions of spinally directed NPY Y1 agonists.

Our results establish GRP-INs (specifically those that coexpress *Npy1r*) as a mediator of neuropathic allodynia. In contrast with our conclusions, GRP-INs were previously described to be exclusively involved in pruritis and not pain-like behavior (52, 61–63). However, previous *Grp* conclusions were made using a GENSAT BAC transgenic *Grp*^Cre^ mouse line, which only labeled about 25% of DH GRP-INs (40, 62) and prevented the functional interrogation of the entire GRP-IN population. By contrast, in this study we utilized a high-fidelity *Grp*^Cre^ mouse line, which labels approximately 90% of the DH GRP-INs (54). Additionally, for the first time to our knowledge we probed the role of GRP-INs in a chronic pain model (SNI) rather than acute pain modalities (i.e. heat, von Frey, pinprick) (54, 62). With these results, we conclude that GRP-INs are the target of intrathecal Y1 agonists to inhibit SNI-induced mechanical and cold hypersensitivity.

**Figure 7:**
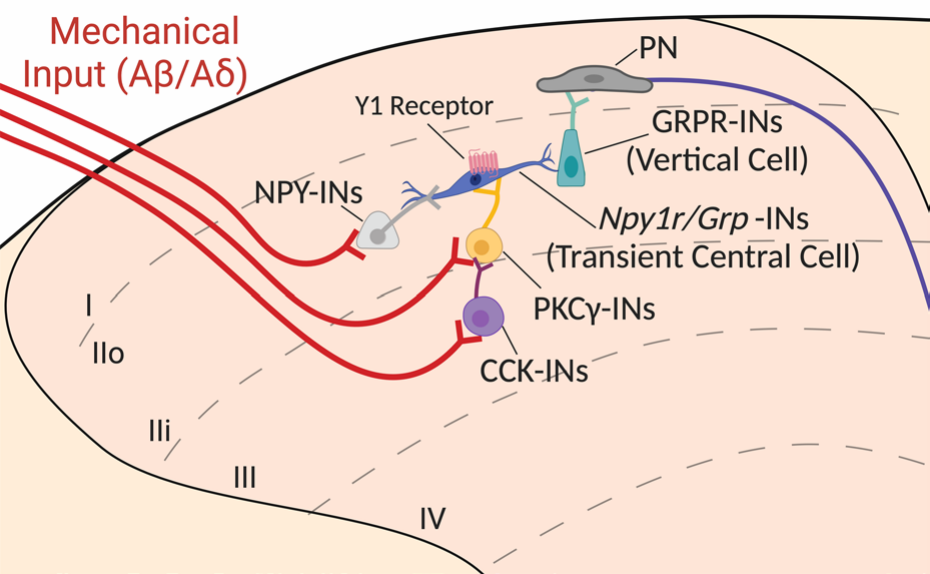
Schematized model for NPY Y1 agonists to inhibit *Grp*/*Npy1r*-INs and dampen neuropathic pain, silencing a key component of the ascending circuit in the dorsal horn that mediates mechanical allodynia. In the context of nerve injury, aberrant hyperexcitation of *Npy1r*/*Grp*-INs may drive allodynia. Exogenous administration of NPY or Y1 agonist binding to the G_i_-coupled NPY Y1 receptor on *Npy1r*/*Grp*-INs results in cellular inhibition and the abolishment of peripheral nerve injury–induced mechanical allodynia. We posit that NPY Y1 agonists act by inhibiting a key neuron population implicated in the transduction of mechanical allodynia. Briefly, non-noxious mechanical stimuli activate Aß/Aδ myelinated afferents (shown in red) that project into the deeper laminae of the dorsal horn and synapse onto interneurons marked by the expression of CCK (purple) and PKCγ (yellow). Normally, feed-forward inhibition prevents the activation of these interneurons, and as a result light touch is perceived as nonpainful. For example, inhibitory NPY interneurons (light gray) may “gate” *Npy1r*/*Grp*-INs to prevent these neurons from being activated and driving pain-like behaviors. However, in the context of neuropathic pain, feed-forward inhibition is lost, and innocuous light touch inputs activate a theorized dorsally directed microcircuit to allow innocuous mechanical sensory information to be perceived as painful. In this theorized circuit, activated CCK and PKCγ interneurons excite transient central cells (theorized here as *Npy1r*/*Grp*-INs), which in turn synapse onto GRPR-INs (vertical cells), which then activate ascending projection neurons (PNs) that travel via the spinothalamic and spinoparabrachial tracts to be processed via higher order pain centers, such as the lateral parabrachial nucleus. Image is updated from our circuit diagrams previously published under CC BY license (24, 26).

Mechanical allodynia is hypothesized to occur via a polysynaptic DH microcircuit that allows A-fibers to transmit innocuous mechanical input to “pain circuits” (15). Y1-INs and GRP-INs have both been characterized as “transient central cells” (neurons in lamina II outer with a central morphology that discharge action potentials transiently during a depolarizing step; ref. (64) in this circuit (24, 62, 65). We theorize that in the context of nerve injury, GRP-specific Y1-IN transient central cells drive both mechanical allodynia and pathological itch (66–68) via activation of GRPR-INs (Figure 7). Indeed, intrathecal administration of NPY inhibits not only the behavioral signs of neuropathic pain but also chemical and mechanical itch (26, 69, 70). Thus, our results support the idea that neuropathic pain and itch share a spinal circuitry that includes GRP/Y1-INs. Accordingly, a pharmacotherapeutic approach to inhibit GRP/Y1-INs could potentially reduce both chronic neuropathic pain-induced hypersensitivity as well as neuropathic itch (26).

### Net DH excitation/inhibition balance or a subpopulation-specific effect.

In addition to our interpretation that intrathecal Y1 agonists act at GRP-INs to inhibit neuropathic pain, an alternative interpretation relates to the appropriate balance of excitation and inhibition (E/I) in the DH, which is critical for appropriate somatosensory processing (2). Peripheral nerve injury produces an enduring shift in the E/I balance of the DH toward an excitatory and pro-nociceptive state (13, 71–76); consequently, inhibition of pain-facilitatory excitatory Y1-INs could restore E/I balance and dampen pathogenic hyperexcitation. In this scenario, it is the total number of glutamatergic pro-nociceptive Y1-INs that are inhibited that is critical for the reduction of neuropathic pain. Therefore, loss of *Npyr* in the *Grp*/*Npy1r*-INs, the largest subpopulation of Y1-INs (~60%) in the superficial DH (Figure 2), might prevent intrathecal NPY from being able to inhibit enough pro-nociceptive Y1-INs to reduce neuropathic hypersensitivity. While this alternative interpretation may be valid, there is evidence to the contrary. For example, inhibition of the larger and predominately excitatory calretinin DH interneuron population (~30% of all superficial dorsal horn neurons are calretinin-immunoreactive, ref. 77; as opposed to ~25% expressing *Npy1r*, Figure 1) did not reduce peripheral nerve injury–induced allodynia (14). Therefore, we believe that neuronal subpopulation specificity, rather than total neuronal number, underlies the loss of Y1 agonist–induced antiallodynia following conditional knockout of *Npy1r* in GRP-INs.

### Evolutionary conservation of Y1-IN subpopulations.

NPY binding sites were previously reported in the superficial laminae of the DH of mouse, rat, and monkey (29, 34, 78–82). Here, using FISH, we expanded these results to demonstrate Y1-IN subpopulations are conserved across the rodent, rhesus macaque, and human (Figure 3). This evolutionary conservation supports their translational relevance. One caveat is that we evaluated human cervical spinal cord as opposed to lumbar spinal cord in macaque and mouse, and we cannot rule out variance in RNA expression between cervical and lumbar spinal cord. However, a single-nucleosome RNA-sequencing database has recently emerged for the adult human lumbar spinal cord (82), and our FISH results obtained in cervical tissue recapitulate many of the single-cell results. Interestingly, in contrast to the mouse, RNA sequencing showed that *NPY1R* is broadly expressed in human dorsal horn across all glutamatergic subpopulations (Ex-Dorsal 1–12) (Supplemental Figure 4). However, like the mouse, specific subpopulations coexpress *CCK* (most densely Ex-Dorsal 2 and 4) or *GRP* (most densely Ex-Dorsal 11–12) (Supplemental Figure 4). One stark contrast with the mouse and macaque is that *NPFF* expression was not detected in the human single-nucleosome RNA-sequencing database (Supplemental Figure 4) (82). However, the lack of *NPFF* expression result further supports the integrity of our cervical FISH results (Figure 3H) and reveals the sparse to nondetectable expression of *NPFF* in the adult human spinal cord DH. Importantly, the *GRP*/*NPY1R*-IN population, the subpopulation that we believe is fundamental to the manifestation of neuropathic pain, is conserved in the macaque and human spinal cord.

### NPY Y1/GRP interneurons — a new therapeutic target?

A rich 30-year history of preclinical research implicates NPY at the spinal cord as a potent inhibitor of acute and chronic pain (26), thereby prompting further investigation of Y1-selective therapeutics. Future investigations might test spinally directed NPY Y1 agonists in increasingly more translatable animal models (i.e., macaque, ref. 83) to perform pain behavioral testing as well as side effect profiling analyses. We are optimistic that Y1 agonism will safely and potently reduce the behavioral signs of neuropathic pain in larger mammalian species, and ultimately, humans with chronic pain. Further, our results suggest that *GRP*/*NPY1R*-INs are particularly well positioned in the spinal cord to modulate clinical neuropathic pain and thus represent a promising and refined therapeutic target for future drug development. In fact, the human RNA-sequencing data suggest that *NPY1R* expression is rather nonspecific across excitatory interneurons in humans, and a *GRP*/*NPY1R*-selective approach may therefore be optimal.

If *GRP*/*NPY1R*-INs are indeed a specific neuronal subpopulation within the human spinal cord that play a crucial role in pain transmission and perception, then the next challenge is to target them effectively and specifically with the delivery of therapeutic agents. A promising future approach is promoter-specific gene therapy (84). Promoters are DNA sequences that determine when and where a gene is expressed. Thus, viral vectors, nanoparticles, or CRISPR/Cas9 gene-editing techniques can be used to introduce *GRP* promoter-specific therapeutic genes into DH neurons, allowing functional modifications to reduce pain signals. The human single-nucleosome RNA sequencing suggests that *NPY1R* is broadly expressed across all excitatory neuronal subpopulations and therefore may not represent a precise therapeutic target. Rather, by using the promoter-specific gene therapy approach, we may specifically target the smaller *GRP* neuronal population and thereby minimize potential side effects of targeting all *NPY1R* neurons. For example, virally expressed genes could produce proteins that modulate pain signaling, reduce neuronal hyperexcitability, or enhance endogenous pain relief mechanisms. Promoter-specific gene therapy for pain is a promising avenue in the development of novel and effective treatments for chronic pain conditions. Long-term safety and efficacy will need to be thoroughly assessed through preclinical and clinical studies to determine its full potential and applicability in clinical settings.

## Methods

### Animals

Adult C57BL/6NCrl (Charles River, 027), *Npy1r*^loxP/loxP^ (courtesy of Herbert Herzog, Garvan Institute, Sydney, New South Wales, Australia; ref. 53), *Grp*^Cre^ (courtesy of Zhou-Feng Chen; Department of Anesthesiology, Washington University School of Medicine, St. Louis, Missouri, USA ref. 85), *Cck*^Cre^ (Jackson Laboratory, 012706), and *Npff*^Cre^ (see below; ref. 86) mice were group-housed; provided access to food and water ad libitum; and maintained on a 12-hour light/12-hour dark cycle (lights on at 7 am) in temperature- and humidity-controlled rooms. Male and female mice were used in all experiments. Although we were not powered to detect significant sex differences, no major/obvious trends in sex differences were observed; means from both sexes were pooled.

The *Npff*^Cre^ knockin mouse line (86) was generated by Taconic Biosciences GmbH (Leverkusen, Germany), using a conventional embryonic stem cell targeting strategy and homologous recombination. Briefly, the sequence for the T2A peptide and the open reading frame of improved Cre recombinase (iCre) were inserted between the last amino acid and the translation termination codon in exon 3 of the NPFF gene. A positive selection marker (puromycin resistance) flanked by FRT sites was removed by crossing *Npff*^Cre^ mice with germline Flpe mice. The presence of the T2A sequence should result in cotranslational cleavage between the NPFF and iCre proteins, resulting in coexpression of both proteins, under control of the Npff promoter.

### Pharmacological testing

[Leu^31^, Pro^34^]-NPY (human, rat) (TOCRIS catalog 1176) was diluted in 0.9% sterile sodium chloride (Medline catalog 63323-186-10) and stored at –20°C. Intrathecal injections of [Leu^31^, Pro^34^]-NPY were performed in lightly restrained, unanesthetized mice. Briefly, a 30G needle attached to a Hamilton microsyringe was inserted between the L5/L6 vertebrae at the cauda equina, puncturing the dura (confirmed by presence of reflexive tail flick). We then injected a 5 μL volume of saline or [Leu^31^, Pro^34^]-NPY (10 μg/5 μL). Animals were injected twice using a crossover design with 3 to 7 days of separation between the 2 injections. For example, animals receiving saline for the first injection received [Leu^31^, Pro^34^]-NPY for the second, and animals receiving [Leu^31^, Pro^34^]-NPY for the first injection received saline for the second. In all cases, group means of saline and [Leu^31^, Pro^34^]-NPY did not differ on either injection day and so were combined for final analysis.

CNO (TOCRIS catalog 4936) was diluted in 0.9% sterile sodium chloride and stored at room temperature. Intraperitoneal injections of CNO (3 mg/kg) or saline were performed in lightly restrained, unanesthetized mice with a 27G permanently attached needle BD Tuberculin Syringe (catalog 305620). Animals were injected using a crossover design with at least 3 days of separation between saline and CNO injections. In all cases, group means of saline and CNO did not differ on either injection day and so were combined for final analysis.

### Surgeries

#### SNI.

SNI was performed as previously described (23, 24). Briefly, mice were anesthetized with inhaled isoflurane (5% induction and 2% maintenance), and the left hind limb was shaved with trimmers and asepticized with 70% ethanol and a ChloraPrep swabstick (BD catalog 260100). A small incision was made in the skin of the hind left leg, and the underlying muscle was spread via blunt dissection to expose the underlying branches of the sciatic nerve. The peroneal and tibial nerves were then ligated with 6-0 silk sutures (Butler Schein Animal Health catalog NC0049524) and transected while carefully avoiding the sural nerve. The muscle tissue was then loosely sutured with 5-0 vicryl sutures (Med Vet International catalog 50-118-0847), and the skin was closed with 9 mm wound clips (Braintree Scientific catalog NC9281117). Topical triple antibiotic ointment (Neosporin Neomycin Sulfate/Bacitracin Zinc/Polymycin Ointment; Hanna Pharmaceutical Supply Co. catalog NC0100117) was applied to the wound. Wound clips were removed 7–10 days postsurgery, and behavioral experiments began 14 days after surgery.

#### Intraspinal AAV injections.

Mice were anesthetized with inhaled isoflurane (5% induction and 2% maintenance), and the skin of the back was shaved with trimmers and asepticized with 70% ethanol and a ChloraPrep swabstick. A midline incision was carefully made to allow visualization of the underlying L1 vertebrae. The L1 vertebrae was then removed by laminectomy, exposing the L4 segment of the spinal cord. A glass microelectrode was inserted into 3 separate locations along the rostral caudal axis of the L4 segment: in the middle and near the boundary with L3 and L5. At each injection site, the glass microelectrode was lowered to a depth of 250 μm below the dura using a stereotaxic frame (David Kopf Instruments Model 940). A total of 333.3 nL of virus (AAV8-hSyn-DIO-mCherry, Addgene catalog 50459-AAV8, or AAV8-hSyn-DIO-hM4Di-mCherry, Addgene catalog 44362-AAV8) was slowly injected into each of the 3 spots (5 nL/s) using a Nanoject III (Drummond catalog 3-000-207) with a 3-minute wait time after completion of each injection to permit adequate infusion. The lassimus dorsi was sutured with 5-0 nylon sutures to protect the exposed spinal cord, and the overlying skin was closed with 9 mm wound clips (Braintree Scientific catalog NC9281117). Topical triple antibiotic ointment (Neosporin Neomycin Sulfate/Bacitracin Zinc/Polymycin Ointment; Hanna Pharmaceutical Supply Co. catalog NC0100117) was applied to the wound. Ethiqa XR buprenorphine extended-release injectable suspension (Fidelis Pharmaceuticals catalog FP-001 072117, 3.25 mg/kg, subcutaneous injection) was utilized as a postoperative analgesic. Behavioral experiments began 21 days after surgery.

### Behavioral testing

#### Mechanical withdrawal threshold.

Testing was performed as previously described (24, 87, 88). Mice were habituated to plexiglass chambers with opaque walls (15 × 4 × 4 cm) on a raised wire mesh platform for 30–60 minutes 1 day before and immediately prior to behavioral testing. Testing was performed using a calibrated set of logarithmically increasing von Frey monofilaments (Stoelting catalog 58011) that range in gram force from 0.007 to 6.0 g. Beginning with a 0.4 g filament, these were applied perpendicular to the lateral hind paw surface with sufficient force to cause a slight bending of the filament. A positive response was denoted as a rapid withdrawal of the paw within 4 seconds of application and was followed by application of the next lower filament. A negative response was followed by application of the next higher filament. An up-down method (89) was used to calculate the 50% withdrawal threshold for each mouse.

#### Cold withdrawal duration.

Immediately following von Frey testing, cold sensitivity testing was performed on mice in the same plexiglass chambers on a raised wire mesh platform. Using a syringe connected to PE-90 tubing, flared at the tip to a diameter of 3.5 mm, we applied a drop of acetone (VWR catalog BDH1101-1LP) to the lateral side of the hind plantar paw (sural nerve innervation territory). Surface tension maintained the volume of the drop to ~10 μL. The duration of time the animal lifted or shook its paw was recorded for 30 seconds. Three observations were averaged.

#### Hind paw brush for Fos.

To produce *Fos* activation, a light-touch stimulation protocol was initiated on the ipsilateral hind paw of SNI mice 14 days after nerve injury. Mice were anesthetized with isoflurane (5% induction, 2% maintenance), and the lateral surface of the left hind paw was gently stroked in the longitudinal plane with a cotton-tipped applicator for 3 seconds every 5 seconds, for 5 minutes. After an additional 60-minute awake and freely moving wait time in their home cage, mice received an overdose injection of sodium pentobarbital (Fatal-Plus, Vortech catalog 9373, 0.25 mL, intraperitoneal injection) and were transcardially perfused.

### FISH (RNAscope)

#### Mice.

Mice were transcardially perfused with ice-cold 1× PBS followed by 10% buffered formalin. Spinal cords were extracted via blunt dissection, postfixed in 10% formalin (2–4 hours), and then placed in 30% sucrose at 4°C until the tissue sank (48–72 hours). L3 and L4 were sectioned (20 μm) on a vibrating microtome (Epredia), floated, mounted on Fisherbrand Superfrost plus microscope slides (Thermo Fisher Scientific), and air-dried overnight at room temperature. FISH pretreatment began with a 10-minute xylene bath, 4-minute 100% ethanol bath, and 2-minute RNAscope H_2_O_2_ treatment. Next, the FISH protocol for RNAscope Multiplex Fluorescent Detection Kit v2 Assay (Advanced Cell Diagnostics catalog 323100) was followed for hybridization to marker probes described in Table 1. Signal amplification was carried out using TSA Fluorescein (PerkinElmer catalog NEL701A001KT), Cyanine 3 (PerkinElmer catalog NEL704A001KT), and Cyanine 5 (PerkinElmer catalog NEL705A001KT) reagents at a 1:1,000 dilution. Slides were then coverslipped with VECTASHIELD HardSet Antifade Mounting Medium with DAPI (VECTOR Laboratories catalog H-1500-10) or processed for mCherry immunohistochemistry.

#### Rhesus macaques.

Two rhesus macaques (male, 4 years at time of death; and female, 4 years at time of death) were provided by David Lewis (University of Pittsburgh) and cared for under the guidelines of the NIH and as approved by the Institutional Animal Care and Use Committee of the University of Pittsburgh. No prior manipulations to the spinal cord were conducted in the macaques. At the time of tissue harvest, macaques were perfused with artificial cerebrospinal fluid, and L5–L7 spinal cord tissue was removed, placed in OCT, and immediately frozen on dry ice. Tissue sections were cut 20 μm thick using a cryostat and mounted onto Superfrost-charged slides, then stored at –80°C. After a 30-minute fixation step with cold 4% paraformaldehyde, the fresh-frozen FISH protocol for RNAscope Multiplex Fluorescent Detection Kit v2 Assay (Advanced Cell Diagnostics catalog 323100) was followed for hybridization to the marker probes described in Table 1. Signal amplification was carried out using TSA Fluorescein, Cyanine 3, and Cyanine 5 reagents (1:1500; Akoya Biosciences). All sections were costained for DAPI and coverslipped at the end of the assay.

#### Human donor spinal cord tissue.

Two human cervical spinal cord tissues were obtained freshly frozen from the NeuroBioBank, NIH (project no. 063772), and provided by Jill Glausier (University of Pittsburgh). All available evidence indicated that these individuals (male, 45 years at time of accidental death; female, 44 years at time of natural death) were not afflicted with any major psychiatric or neuropathological illnesses at the time of death. All procedures were approved by the Committee for the Oversight of Research and Clinical Training Involving Decedents at University of Pittsburgh, Pittsburgh, Pennsylvania. The fresh-frozen FISH protocol for RNAscope Fluorescent Multiplex Reagent Assay (Advanced Cell Diagnostics catalog 320850) was followed for hybridization to the marker probes described in Table 1. Briefly, 16 μm–thick, fresh-frozen human spinal cord sections were fixed in 4% paraformaldehyde, dehydrated, treated with protease for 15 minutes, and hybridized with gene- and species-specific probes.

### Immunohistochemistry

Immediately following the RNAscope V2 protocol, mouse spinal cord sections were washed 3 times in PBS and then pretreated with blocking solution (3% normal goat serum and 0.3% Triton X-100 in PBS) for 1 hour. Sections were then incubated overnight on a slow rocker at 4°C in blocking solution containing the primary antibody (1:2,000, Anti-mCherry; Invitrogen catalog M11217). The sections were washed 3 times in 1× PBS and then incubated in secondary antibody (1:1,000, Alexa Fluor 568 Goat anti-Rat; Invitrogen catalog A11077) for 60 minutes. Finally, sections were washed in 3 times in 1× PBS and then 2 times in 0.01 M phosphate buffer without saline before mounting the tissue to Fisherbrand Superfrost Plus microscope slides (Thermo Fisher Scientific catalog 12-550-15) and coverslipping with VECTASHIELD HardSet Antifade Mounting Medium with DAPI (VECTOR Laboratories catalog H-1500-10).

### Microscopy and quantification

All images were captured on a Nikon Eclipse Ti2 microscope using a 20× or 40× objective and analyzed using NIS-Elements Advanced Research software v5.02 (Nikon). Cells with at least 3 puncta associated with a DAPI^+^ nucleus were considered positive. In mouse, positive cells within the superficial 100 μm of the DH were quantified in 3–5 sections and averaged to yield 1 data point for each mouse. In macaque,s positive cells within the substantia gelatinosa (translucent area in the superficial DH) were quantified, and 1 spinal cord section represents 1 individual data point. Intense lipofuscin prevented quantification in the human spinal cord.

### Masking procedures

Experimenter masking (blinding) was employed to promote research rigor. In all cases the experimenter was masked to drug treatments and transgenic mouse genotype. Intrathecal injections were performed by a laboratory colleague, thus providing complete anonymity of drug agent for each animal. In the chemogenetic experiments the experimenter was masked both at the time of surgery to virus (DREADD vs. control) and at the time of behavior to drug agent (CNO vs. saline). The code key was kept hidden in a notebook and not revealed until after the completion of each experiment.

### Data presentation

Graphs and images were created in GraphPad Prism 9.0, meta-chart.com (Venn Diagram Maker Online), Adobe Illustrator 26.3, or BioRender.com.

### Statistics

Data were graphed and analyzed using GraphPad Prism 9.0 using unpaired 2-tailed *t* tests for the data of Figure 4, B and C; 2-way ANOVA (mRNA expression × stimulation) followed by a Holm-Šídák test when appropriate for the data of Figure 4E; 3-way RM ANOVA (virus × side × drug as the repeated measure) for the data of Figure 5, D–G; or 3-way RM ANOVA (genotype × drug × time as the repeated measure) followed by Tukey’s test when appropriate for the data of Figure 6, H and I. Statistical significance was determined as *P* < 0.05. Data are presented as mean ± SEM.

### Study approval

All procedures were consistent with the NIH’s *Guide for the Care and Use of Laboratory Animals* (National Academies Press, 2011), followed the guidelines for the treatment of animals of the International Association for the Study of Pain, and were approved by the Institutional Animal Care and Use Committee and the Committee for the Oversight of Research and Clinical Training Involving Decedents at University of Pittsburgh, Pittsburgh, Pennsylvania.

### Data availability

All raw data are included in the Supporting Data Values file. Images are available from the corresponding author upon reasonable request.

## Author contributions

TSN, BKT, and AJT designed research; TSN, HNA, PB, PP, EN, CMA, and DFSS performed research; TSN, HNA, PB, PP, BKT, and AJT analyzed data; TSN and BKT wrote the manuscript with contributions from HNA, AJT, and RPS; TSN, HNA, AJT, SER, and BKT received funding for the project; and BKT supervised the overall project.

## Supplementary Material

Supplemental data

Supporting data values

## Figures and Tables

**Table 1:**
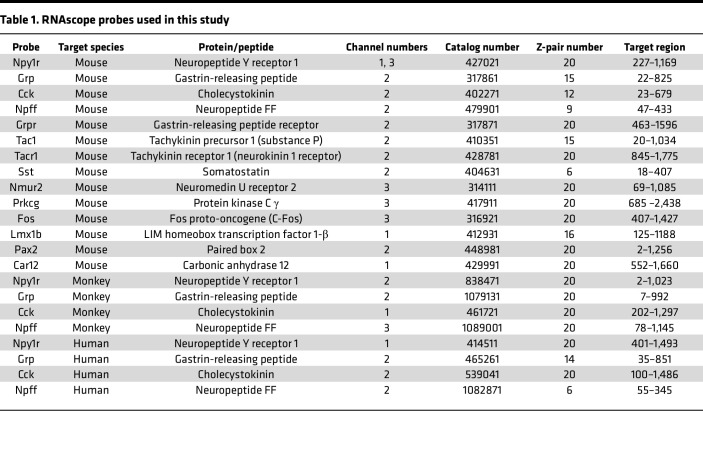
RNAscope probes used in this study

## Version 1 10/12/2023

In-Press Preview

## Version 2 11/22/2023

Electronic publication
