# Supplementary material for: Alleviation of neuropathic pain with neuropeptide Y requires spinal Npy1r interneurons that coexpress Grp
Source: JCI Insight. 2023 Nov 22;8(22):e169554. doi: 10.1172/jci.insight.169554 (PMC10721324; doi:10.1172/jci.insight.169554)
Supplement: Supplemental data [file jciinsight-8-169554-s009.pdf]

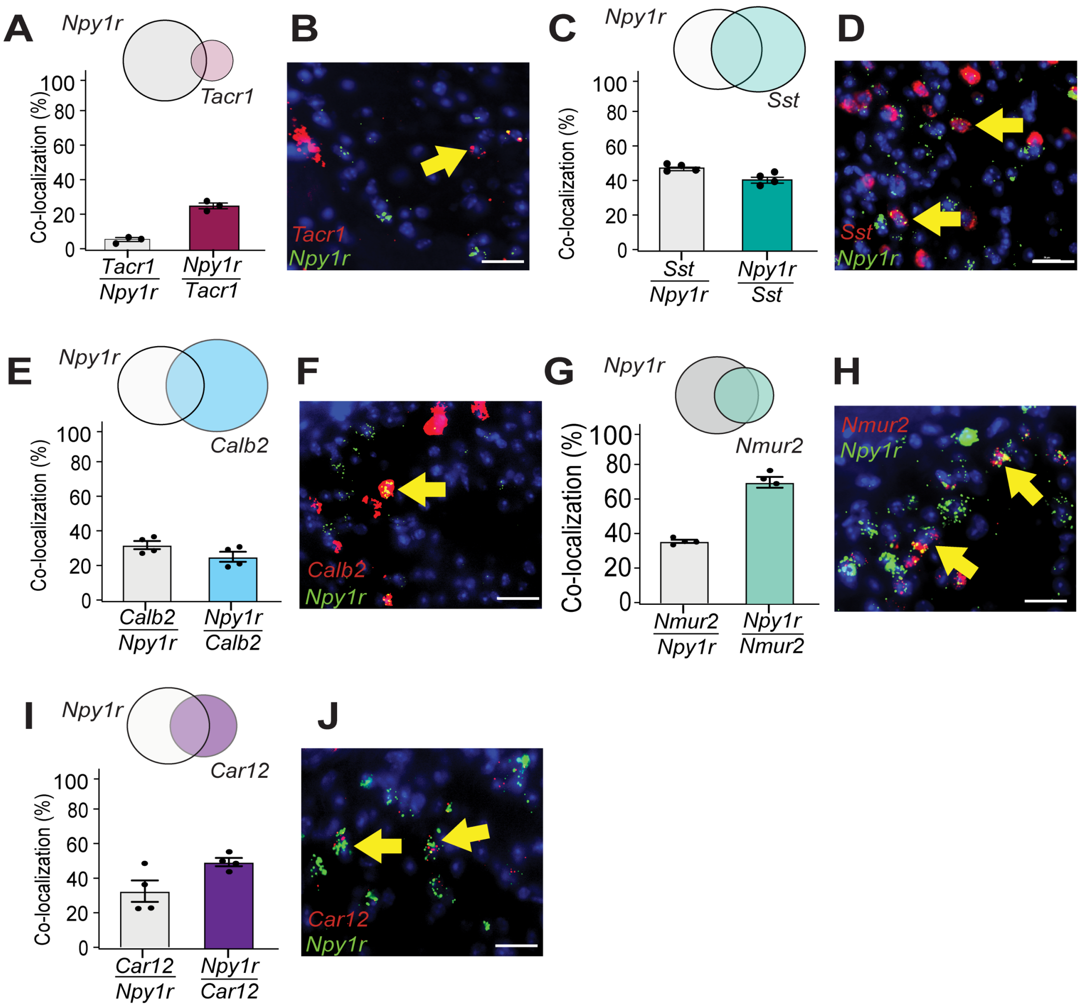

Supplemental Figure 1. Co-localization of *Npy1r* with additional mRNAs

(**A-B**) *Npy1r* in laminae I-II co-localizes with *Tacr1* (*Tacr1*/*Npy1r* - 6.94 ± 1.11%; *Npy1r*/*Tacr1* - 28.34 ± 1.71%), (**C-D**) *Sst* (*Sst*/*Npy1r* - 49.26 ± 0.98%; *Npy1r*/*Sst* - 42.98 ± 1.67%), (**E-F**) *Calb2* (*Calb2*/*Npy1r* - 33.96 ± 2.42%; *Npy1r*/*Calb2*- 24.94 ± 2.81%), (**G-H**) *Nmur2* (*Nmur2*/*Npy1r* - 37.04 ± 0.94%; *Npy1r*/*Nmur2*- 72.50 ± 3.24%), and (**I-J**) *Car12* (*Car12*/*Npy1r* - 32.83 ± 6.33%; *Npy1r*/*Car12*- 50.42 ± 2.39%) (n=3-5 mice/group).

Each data point indicates the average of 2-4 quantified sections/mouse. Scale bars: 25 µm.

Yellow arrows indicate co-localization. Data shown as mean ± SEM.

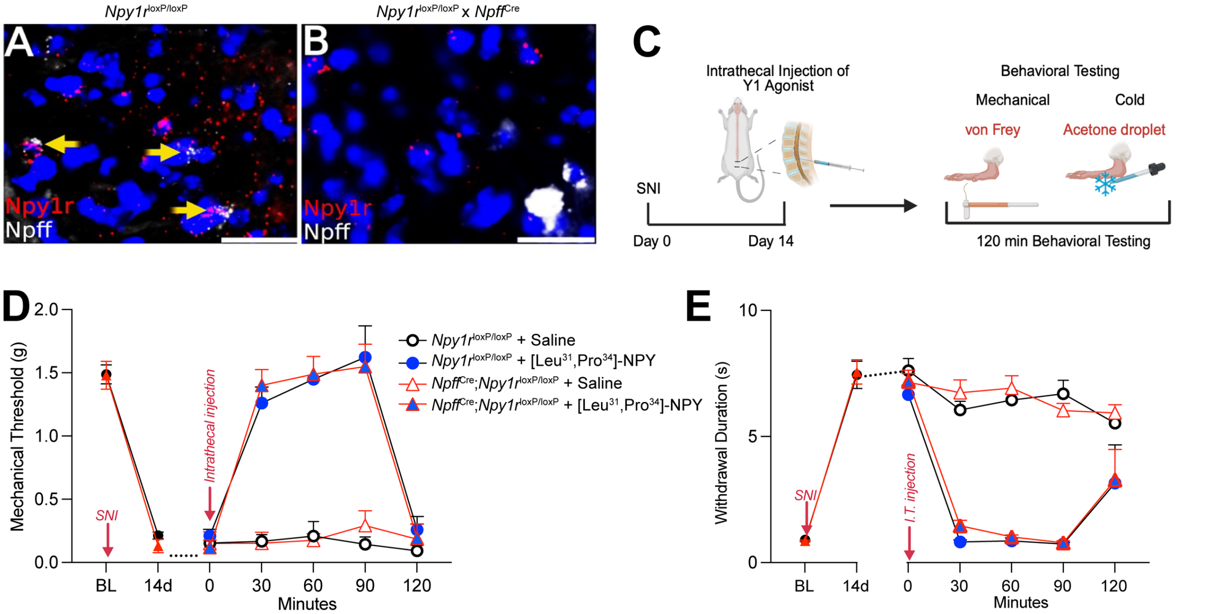

Supplemental Figure 2. *Npff*/*Npy1r*-INs do not contribute to the inhibition of nerve injury-induced mechanical and cold allodynia by a Y1 agonist.

(**A-B)** Confirmation of conditional deletion of *Npy1r*. Fluorescence *in situ* hybridization of sections of the lumbar spinal cord demonstrate that *Npy1r*$^{loxP/loxP}$ mice contain DH neurons that co-express *Npy1r* and *Npff*. Conversely, *Npy1r*$^{loxP/loxP}$;*Npff*^{Cre} mice lack expression of *Npy1r* in *Npff*-expressing neurons. Yellow arrows indicate co-localization. Scale bars: 25 μm.

(**C**) Experimental timeline for SNI, intrathecal pharmacology, and mechanical (von Frey) and cold (acetone droplet withdrawal) behavioral testing.

(**D**) [Leu^{31}, Pro^{34}]-NPY abolished SNI-induced mechanical allodynia in *Npy1r*$^{loxP/loxP}$ and *Npy1r*$^{loxP/loxP}$;*Npff*^{Cre} mice (n=5 mice/group). Three-way RM ANOVA: Time x Genotype x Drug, F (4,64) = 0.5068, P=0.7309).

(**E**) [Leu^{31}, Pro^{34}]-NPY abolished SNI-induced cold allodynia in *Npy1r*$^{loxP/loxP}$ and *Npy1r*$^{loxP/loxP}$; *Npff*^{Cre} mice (n=5 mice/group). Three-way RM ANOVA: Time x Genotype x Drug, F (4,64) = 0.3723, P=0.8276).

Data shown as mean ± SEM.

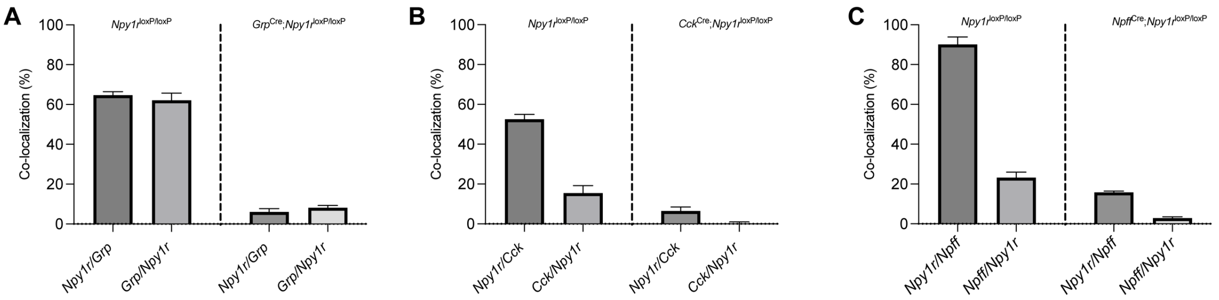

Supplemental Figure 3. Fluorescence *in situ* hybridization confirmation of *Npy1r* knockout in conditional genetic knockout crosses.

Confirmation of conditional deletion of *Npy1r*. Fluorescence *in situ* hybridization in sections of the lumbar spinal cord demonstrate that *Npy1r* is robustly downregulated in *Grp*-expressing neurons from *Npy1r*$^{\text{loxP/loxP}}$;*Grp*^{Cre}, *Cck*-expressing neurons from *Npy1r*$^{\text{loxP/loxP}}$;*Cck*^{Cre}, and *Npff*-expressing neurons from *Npy1r*$^{\text{loxP/loxP}}$;*Npff*^{Cre} mice. (n=1 section each from 3 mice/group). Data shown as mean ± SD.

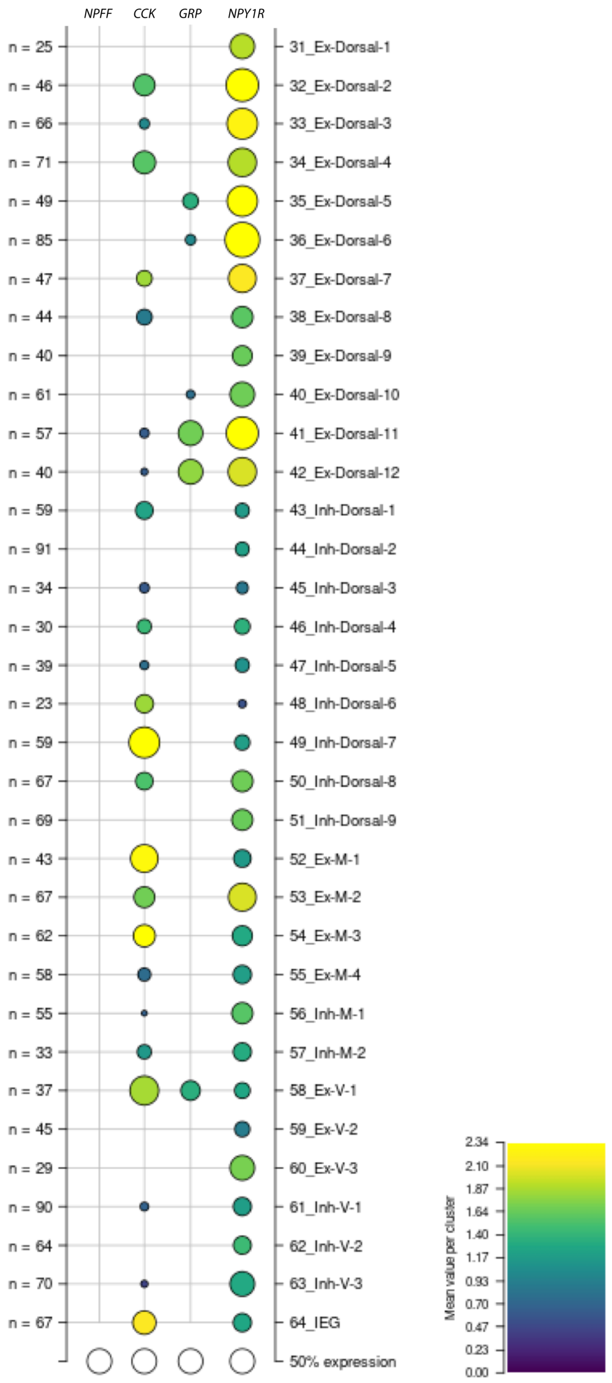

Supplemental Figure 4. Single-nucleosome RNA-sequencing detection of *NPY1R*, GRP, CCK and NPFF in the human lumbar spinal cord.

Dot plot showing the average gene expression for *NPY1R*, *CCK*, *NPFF*, and *GRP* for each human spinal cord cluster identified using single-nucleus RNA sequencing. Data analyzed from https://vmenon.shinyapps.io/humanspinalcord/ stemming from (82).
